# Beyond “Taiji Diagram”: how multimodal metaphor deciphers Yin Xu and Yang Xu through Traditional Chinese Medicine science communication short videos

**DOI:** 10.3389/fpubh.2026.1781678

**Published:** 2026-05-05

**Authors:** Shaoci Wang, Musi Wang, Lay Shi Ng, Azianura Hani Shaari

**Affiliations:** 1 School of Foreign Language, Cangzhou Jiaotong College, Huanghua, China; 2 The Centre for Research in Language and Linguistics, The National University of Malaysia, Bangi, Malaysia; 3 School of Basic Medicine, Tianjin Medical University, Tianjin, China

**Keywords:** Douyin, multimodal metaphor, science communication, Traditional Chinese Medicine, Yang Xu, Yin Xu

## Abstract

**Introduction:**

This study investigates how multimodal metaphors convey two core Traditional Chinese Medicine (TCM) concepts— “Yin Xu” (Yin deficiency) and “Yang Xu” (Yang deficiency)—in short science communication videos on Douyin. Drawing on Conceptual Metaphor Theory, Multimodal Metaphor Theory, and Visual Grammar Theory, this research examines the effectiveness of multimodal representation of TCM concepts in digital health communication.

**Methods:**

This study adopts a quantitative multimodal discourse analysis approach to analyze 15 short videos on Yin Xu and Yang Xu published on Douyin between 2023 and 2024. Using ELAN software, 89 metaphorical units were annotated across four semiotic modes (written signs, pictorial signs, sound, and gesture) to evaluate their performance, distribution, and cross-modal coordination.

**Results:**

Findings indicate that 43.8% of metaphorical mappings exhibit source-target domain inconsistencies, accompanied by insufficient cross-modal coordination and minimal dynamic narrative elements. Written text overwhelmingly dominates the modal distribution at 67.4%, while visual elements remain notably underrepresented at 21.3%. These findings suggest that multimodal inconsistency and visual underutilization may compromise the conceptual coherence of TCM metaphorical communication.

**Discussion:**

In response to these identified shortcomings, this paper presents optimization strategies focused on color-based coding systems, dynamic narrative construction, and enhanced cross-modal integration. By developing a systematic framework for enhancing the precision and coherence of TCM metaphor representation in digital media, this research contributes to the convergence of semiotic analysis, science communication, and cognitive linguistics.

## 1 Introduction

The digital era has transformed the distribution and consumption patterns of health information. Short-video platforms have particularly emerged as influential science communication vehicles, engaging millions through algorithmically curated, visually compelling content (1, 2). Applications like TikTok (known as Douyin in China) now play pivotal roles in public health communication, creating novel pathways for converting sophisticated medical concepts into widely more accessible forms (3, 4). Yet this transformative shift presents notable challenges: the need for simplification to achieve mass accessibility risks undermining scientific precision and nuance, prompting legitimate questions about the reliability and integrity of health-related social media content (5, 6).

Such tensions become especially pronounced when Traditional Chinese Medicine (TCM) is transmitted through digital channels. Throughout and following the COVID-19 pandemic, TCM short videos surged across Douyin, employing multimodal techniques to convey a range of TCM principles (7–9). Previous studies have cautioned that overly reductive multimodal presentations may distort the accuracy of TCM knowledge (10–12). Yet systematic investigations into how multimodal components work together or conflict when representing TCM metaphorical frameworks remain scarce. This research fills that void by analyzing the multimodal strategies used to communicate the fundamental TCM concepts of “Yin Xu” (Yin deficiency) and “Yang Xu” (Yang deficiency), aiming to enhance both the scientific rigor and public accessibility of TCM communication in digital media.

## 2 Research background

Since the COVID-19 outbreak, Traditional Chinese Medicine (TCM) science communication short videos have experienced rapid growth across major Chinese social media platforms, especially Douyin (7, 8). This phenomenon reflects broader shifts in how public seek health information for disease recognition and prevention (13). Such videos have emerged as primary vehicles for converting traditional medical knowledge into broadly comprehensible formats. These productions regularly convey complex theoretical principles—such as Qi, Yin-Yang balance, and syndrome differentiation—using combinations of visual components, voice-over narration, textual annotations, gestural demonstrations, and sound design (14). The compelling nature of this format has driven increased viewership and audience expansion, solidifying short videos as the predominant medium for TCM science communication (6).

Research has recognized these videos’ capacity for promotion and education, demonstrating that multimodal formats boost viewer engagement and understanding (1, 15). Yet, However, numerous researchers caution that such multimodal techniques frequently prioritize esthetic appeal or entertainment over knowledge transmission, compromising the scientific integrity and trustworthiness of TCM discourse (5, 6, 8, 11). This concern intensifies when examining depictions of Yin Xu and Yang Xu—fundamental metaphorical concepts central to TCM’s diagnostic framework for bodily disequilibrium.

In TCM theory, Yin Xu refers to the depletion of nourishing, cooling substances within the body, manifesting as “heat syndromes” such as internal dryness, restlessness, and hot sensations in extremities. Conversely, Yang Xu denotes an insufficiency of vital warmth and metabolic activity, leading to “cold syndromes” such as fatigue, chilliness, and reduced libido (16). Classical texts such as *Huangdi Neijing* (*The Yellow Emperor’s Classic of Medicine*) metaphorically describe Yin Xu as “a lamp without oil,” symbolizing excessive internal heat consuming the body’s remaining fluids, whereas Yang Xu is likened to “a courtyard frozen year-round,” evoking immobility and coldness (17, 18).

**Figure 1:**
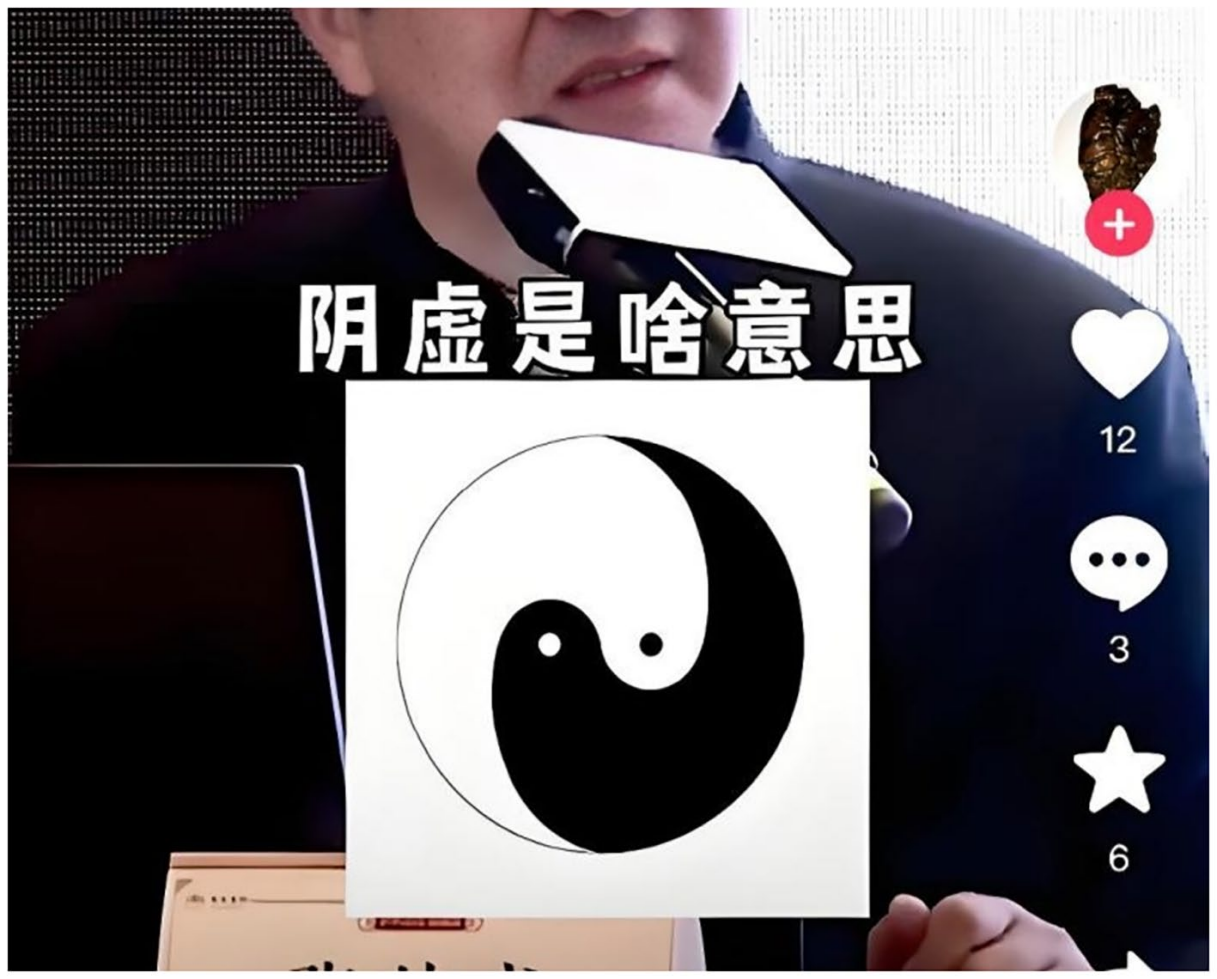
Example of superficial representation of Yin Xu and Yang Xu by “Taiji Diagram”.

Nevertheless, numerous digital presentations distort these principles through excessive dependence on standardized visual conventions—particularly the Taiji Diagram (Yin-Yang symbol)—while failing to coordinate visual or acoustic elements with core metaphorical reasoning. These shallow representations generate conceptual misunderstandings and incorrect connections, theoretically impeding viewer comprehension. Figure 1 demonstrates how the Taiji Diagram inadequately captures the complex pathological dynamics linking Yin Xu and Yang Xu. This overuse of symbolic imagery exemplifies a fundamental challenge in multimodal TCM metaphor presentation: the absence of coherent alignment between conceptual frameworks (pathological processes) and communicative modes (visual, written, acoustic, and kinesthetic elements).

To bridge this research gap, this study investigates the multimodal representation of Yin Xu and Yang Xu in Douyin short videos, aiming to pinpoint areas of inconsistency and establish evidence-based design principles for improving semiotic coherence. Utilizing Conceptual Metaphor Theory, Multimodal Metaphor Theory, and Visual Grammar Theory as analytical frameworks, this research applies quantitative approaches to evaluate the interaction, performance, and synchronization of various semiotic modes. The study pursues three primary objectives: (1) identifying multimodal co-occurrence patterns used to depict Yin Xu and Yang Xu; (2) examining how closely different modes—textual, visual, auditory, and gestural—correspond with TCM’s fundamental metaphorical structure; and (3) formulating design strategies that optimize the precision and coherence of these multimodal presentations.

## 3 Methods

### 3.1 Research design

**Figure 2:**
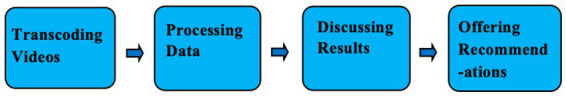
Research workflow of multimodal metaphor analysis.

This study adopted a quantitative multimodal discourse analysis to examine how short science communication videos on Douyin represent the TCM metaphorical pair Yin Xu and Yang Xu (Figure 2). The goal was to explore how multiple semiotic modes—written text, imagery, sound, and gesture—interact in constructing or distorting the conceptual metaphors embedded in TCM theory. The analysis combined both bottom-up empirical annotation and top-down theoretical modeling. The research design was guided by three complementary frameworks: Conceptual Metaphor Theory, emphasizing source-target domain mapping; Multimodal Metaphor Theory, addressing how digital discourse shape metaphor construction; and Visual Grammar Theory, highlighting the role of layout, salience, and color in visual meaning-making.

### 3.2 Theoretical framework

The study’s analytical foundation is grounded in Conceptual Metaphor Theory, Multimodal Metaphor Theory, Visual Grammar Theory.

Conceptual Metaphor Theory posits that abstract concepts are understood through systematic mappings from concrete experiences, known as source-to-target domain relations. For metaphors to function coherently, these mappings must preserve inferential structure and cognitive topology (19).

Multimodal Metaphor Theory extends this notion by emphasizing that metaphoric mappings can occur across modes such as text, image, sound, and gesture. A well-constructed multimodal metaphor ensures cross-modal consistency, meaning that visual and auditory elements reinforce rather than contradict the conceptual mapping established by the verbal mode (20).

Visual Grammar Theory further explains how visual parameters such as salience, composition, and color contribute to meaning construction. In this framework, modal weighting—the relative perceptual importance assigned to each mode—plays a critical role in maintaining interpretive balance (21).

Synthesizing these perspectives, this study develops an operational framework for multimodal analysis, assessing both modal performance (clarity, consistency, and dynamic design) and modal coordination (cross-modal complementarity and temporal alignment). This model underpins the annotation and quantitative analysis procedures detailed in subsequent sections (Table 1).

**Table 1:** The list of metaphors.

Yin Xu (Yin deficiency, 阴虚) vs. Yang Xu (Yang deficiency, 阳虚)		
1	阳虚是寒症	Yang deficiency is manifested in cold syndrome
2	阴虚是热症	Yin deficiency is manifested in heat syndrome
3	阳虚小便清长便溏	Yang deficiency causes clear and profuse urination, loose stools
4	阴虚便干	Yin deficiency causes dry stools
5	阳虚畏寒无力	Yang deficiency causes cold aversion and listlessness
6	阴虚手脚发热坐立不安	Yin deficiency causes hot hands and feet as well as restlessness
7	阳虚性欲弱	Yang deficiency causes low libido
8	阴虚性欲强	Yin deficiency causes high libido
9	阳虚腰膝冷痛	Yang deficiency causes cold pain in the waist and knees
10	阴虚腰膝酸软	Yin deficiency causes soreness and weakness in the waist and knees
11	阳虚性功能下降	Yang deficiency causes decreased sexual function
12	阴虚五心烦热	Yin deficiency causes burning sensation in the five centers (palms, soles, and chest)

### 3.3 Data collection

Fifteen short videos were purposively selected from the Douyin platform (2023-2024). The sample size was determined based on established practices in multimodal metaphor research, where in-depth annotation of metaphorically dense units, rather than statistical representativeness, is prioritized (20, 22, 23). The selected videos yielded 89 metaphorical units, a corpus size comparable to or exceeding those in prior multimodal studies, thereby providing sufficient data for identifying recurrent patterns of modal coordination and inconsistency.

The sample was drawn from a variety of content producers in the Douyin TCM ecosystem including official institutional accounts, professional practitioners, commercial media, and certified organizations, thus reflecting the diversity of real-world TCM science communication. The selection criteria were as follows: (1) Each video explicitly focuses on the TCM concepts Yin Xu and/or Yang Xu; (2) Each includes at least two distinct modes (e.g., written text + imagery, or sound + gesture); (3) Duration does not exceed 3 minutes, and the video has over 1,000 likes, ensuring public engagement; (4) Each features a real practitioner providing spoken narration, serving as the anchoring mode for multimodal analysis. All videos were downloaded in MP4 format and imported into Descript for transcription and timestamp alignment. The transcripts were then verified manually to ensure synchronization between spoken content and visual sequences.

### 3.4 Coding scheme and annotation

The multimodal dataset was annotated using Eudico Linguistic Annotator (ELAN 6.9), following Forceville’s typology of multimodal metaphors (19). Four modes were analyzed—written text (W), pictorial imagery (P), sound (S), and gesture (G)—with voiceover (V) treated as the anchoring mode. Two analytical tiers were established: Tier 1: Modal Performance—evaluating each mode’s accuracy in mapping the source and target domains. Tier 2: Modal Distribution and Coordination—examining co-occurrence, weighting, and temporal alignment across modes. Each multimodal metaphorical unit was annotated using the following codes (Table 2). A total of 89 metaphorical units were annotated and coded according to this framework.

**Table 2:** Coding scheme for multimodal metaphor analysis.

Code	Meaning
[T]	The target domain
[S]	The source domain
[vt]	Voiceover (the target domain)
[vs]	Voiceover (the source domain)
[w]	Written signs
[p]	Pictorial signs
[s]	Sounds
[g]	Gestures
[SNC]	The mode representing the source domain is inconsistent with (vaguely associated with or totally conflicting with) the target domain
[SNS]	The modes representing the source domain appears too fast or imperceptibly
[SND]	The mode representing the source domain is lack of dynamic design
[SQ]	The mode representing the source domain is correct and clear
[N]	The mode representing the source domain is absent
[MR]	The same mode representing the source domain is repeated
[MC]	The modes representing the source domain are conflicting
[MOS]	The modes representing the source domain are asynchronous
[MI]	The mode with high perceptual salience is absent and the modes with low perceptual salience are over-dominance
[wd/pa]	Written sign is over-dominance and pictorial sign is absent
[MCW]	The complementarity of modes representing the source domain is weak
[MCW-r]	≥One mode representing the source domain are redundant replicated
[MCW-v]	≥Two modes representing the source domain exhibit vague or irrelevant mapping
[MSP]	The co-occurrence pattern of the modes representing the source domain

To ensure coding transparency, this study developed explicit decision rules for the primary category [SNC]. Drawing on classical TCM pathology—wherein Yin Xu manifests as heat syndromes and Yang Xu as cold syndromes, two distinct subcategories were identified. “Total conflict” was annotated when a mode triggered a source domain that fundamentally opposed the target domain. Conversely, “vague association” was identified when a mode utilized neutral or ambiguous elements that failed to evoke any meaningful source domain connected to the metaphor (see Table 3 for annotation examples).

**Table 3:** Coding examples for the category [SNC].

Sub-type	Example	Explanation
Total conflict	Blue-colored text for Yin Xu	Yin Xu denotes heat syndrome, but blue visually primes “cold,” directly contradicting the target domain.
Total conflict	Red-colored text for Yang Xu	Yang Xu denotes cold syndrome, but red visually primes “heat,” contradicting the target domain.
Vague association	Black or white text for Yin Xu or Yang Xu	Neutral colors provide no metaphorical cues, failing to activate either heat or cold associations

These coding decision rules were grounded in classical TCM interpretations of Yin Xu and Yang Xu as documented in the *Huangdi Neijing* and subsequent authoritative texts (detailed in Section 2). To verify the metaphorical accuracy of the coding framework, all annotations were cross-referenced with canonical TCM literature and subjected to validation discussions within the research team, which included members with formal training in TCM theory. In cases of ambiguity, TCM practitioners from affiliated institutions were consulted for additional expertise. This multi-layered validation approach ensured the coding integrated both rigorous semiotic analysis and specialized domain knowledge from TCM.

### 3.5 Reliability and data processing

All metaphorical units were independently coded by two trained analysts with expertise in TCM discourse and multimodal analysis. Cohen’s *κ* was calculated to measure inter-coder reliability, yielding a coefficient of 0.86, which demonstrates strong agreement between coders. The analytical approach focused on uncovering patterns and developing descriptive insights into modal distribution, performance, and coordination. Descriptive statistics (frequencies and proportions) were calculated using Microsoft Excel 365, which facilitated the creation of frequency tables, cross-modal co-occurrence maps, and performance distribution charts.

The analytical framework comprised three sequential stages. First, frequency analysis identified dominant co-occurrence patterns among modes. Second, performance evaluation quantified the proportion of inconsistencies, omissions, and clear mappings within the corpus. Third, cross-modal visualization generated interaction charts to reveal structural relationships among modes. This hybrid computational-manual methodology balanced quantitative rigor with interpretive nuance, enabling systematic investigation of how multimodal configurations affect the conceptual coherence of TCM metaphors.

## 4 Results

This section presents the results of the multimodal annotation and quantitative analysis. The findings are organized into three parts: (1) modal co-occurrence patterns, (2) modal performance and distribution, and (3) cross-modal coordination.

### 4.1 Modal co-occurrence patterns

A total of 89 multimodal metaphorical units were identified across the 15 selected Douyin videos. The analysis revealed distinct co-occurrence tendencies among the modes.

**Figure 3:**
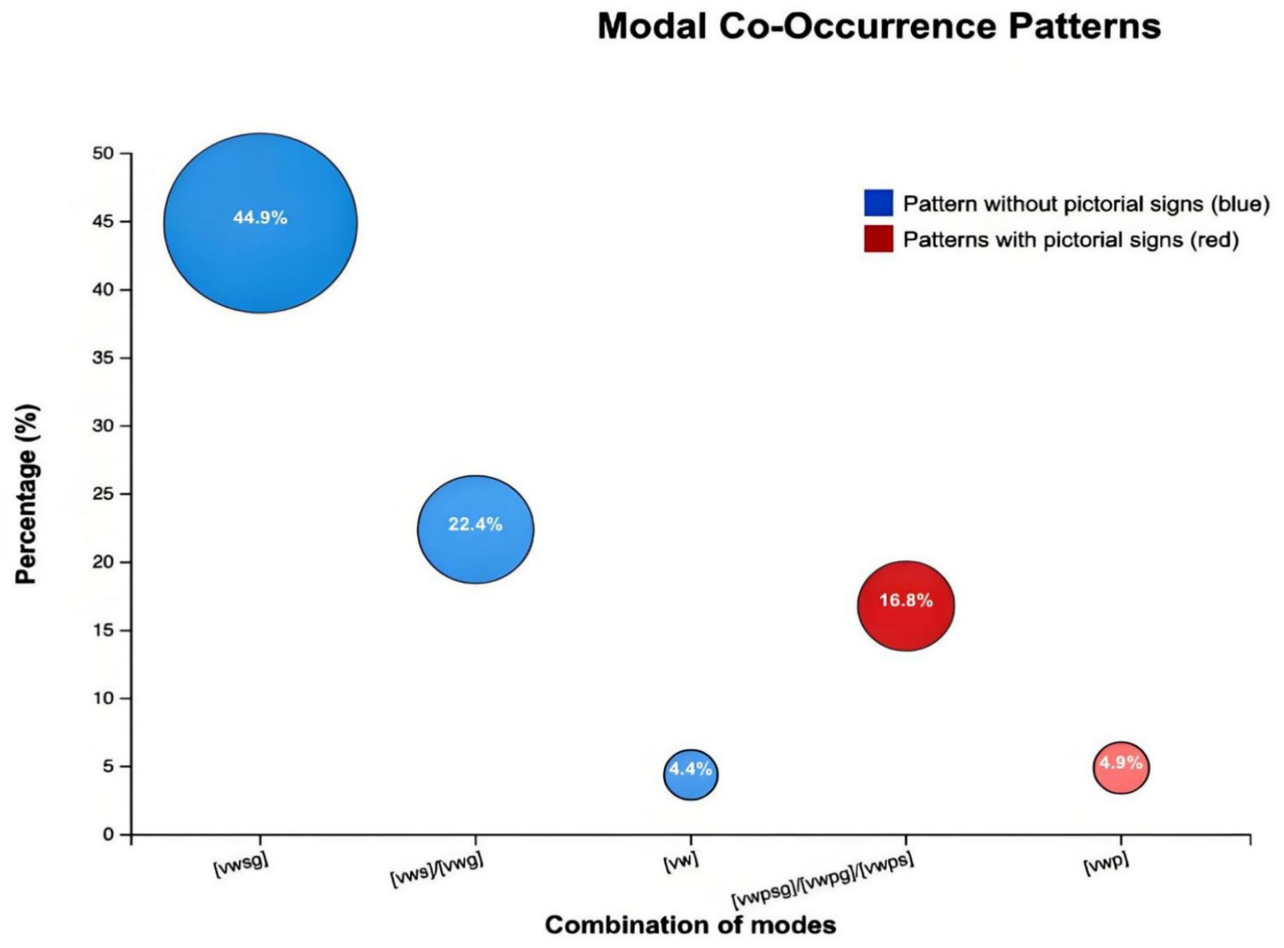
Modal co-occurrence patterns in TCM short videos.

Voiceovers and written text were the most dominant combination, accounting for 67.4% of all metaphorical units, while purely verbal combinations (voiceover only) constituted only 4.4%. By contrast, pictorial signs appeared in merely 21.3% of the units, indicating an overall underutilization of visual imagery. Among the observed configurations, [VWSG] (voiceover + written text + sound + gesture) was the most frequent, followed by [VWS] and [VWG], which together formed over two-thirds of all combinations. More complex integrations including pictorial elements ([VWPSG], [VWPG], [VWPS]) represented only 16.8% of the total, underscoring the imbalance between linguistic and visual modes. These data indicate that while multimodality is present, it remains largely text-centered, with other modes serving supplementary rather than complementary roles (Figure 3).

### 4.2 Modal performance and distribution

**Figure 4:**
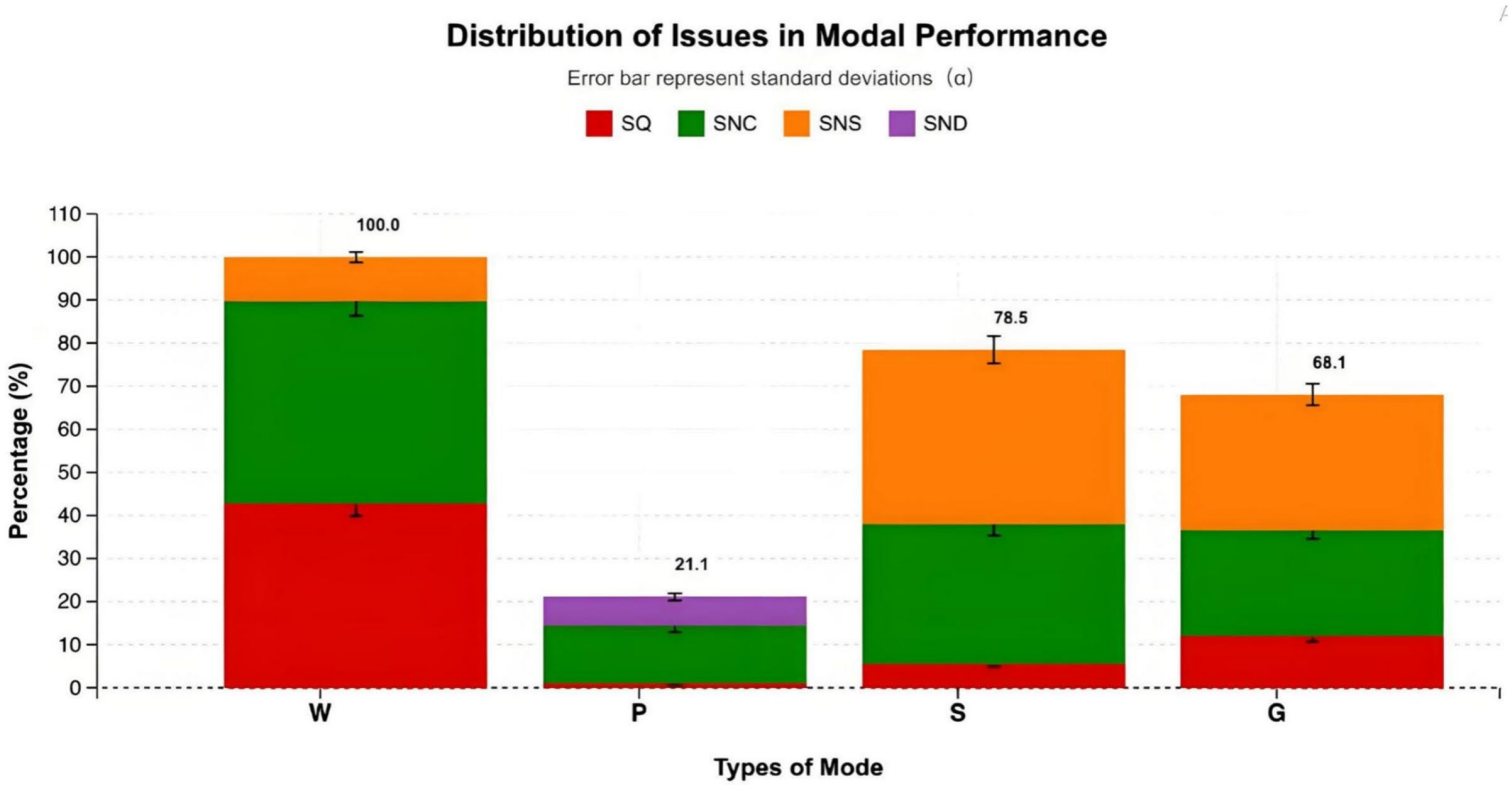
Modal performance and accuracy distribution across modes.

The performance evaluation revealed significant variations in mapping clarity and modal accuracy. Written text appeared in nearly all metaphorical units but demonstrated the highest inconsistency rate. Out of all text-involving instances, 43.8% were coded as inconsistent or conflicting with the target domain ([SNC]), and 41.5% were categorized as clear and correct ([SQ]). Additionally, 13 units exhibited brief or imperceptible text exposure ([SNS]), suggesting that excessive text density or rapid presentation may reduce the clarity of the metaphorical mapping. Pictorial imagery showed a relatively low utilization rate and was often static or incomplete. Only 13.4% of pictorial elements were inconsistent with the target domain ([SNC]), but 6.7% lacked dynamic representation ([SND]), reducing their interpretive power. Auditory elements displayed notable weaknesses. Nearly 40.4% of sound cues were coded as imperceptible ([SNS]), and 32.5% were inconsistent with the verbal message ([SNC]). Only five auditory instances (5.6%) demonstrated accurate metaphorical correspondence. Gestures performed slightly better but still showed limited mapping precision: 24.7% were inconsistent ([SNC]) and 31.4% too brief ([SNS]) to contribute to the accessibility of the intended meaning. Only 12% were rated as clear and correct ([SQ]) (Figure 4).

Overall, these results reveal that written text and voiceovers are the predominant modes in the targeted short videos, but both exhibit frequent inconsistencies. Non-verbal modes—particularly sound and gesture—contribute minimal metaphorical precision, constrained by brevity, redundancy, and weak dynamic synchronization with other modes.

### 4.3 Cross-modal coordination

**Figure 5:**
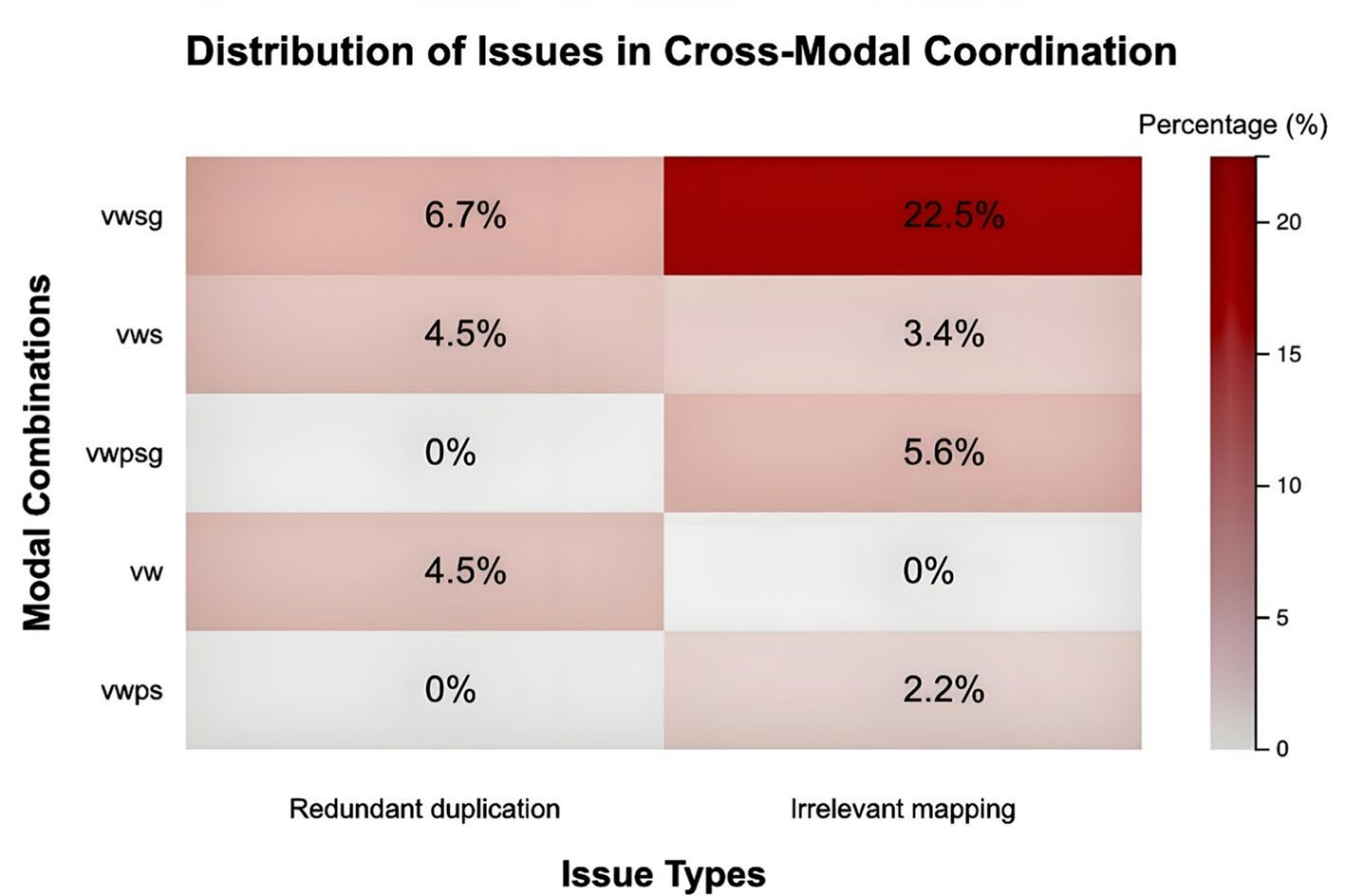
Cross-modal coordination and redundancy patterns.

Analysis of modal coordination patterns highlighted persistent issues in complementarity and synchronization. While overall cross-modal asynchrony ([MOS]) remained low at 6.7%, nearly half of all metaphorical units (≈50%) displayed weak cross-modal collaboration. Within these, 32 units exhibited vague or irrelevant mappings ([MCW-v]), and 14 units showed redundant duplication ([MCW-r]). The severity of coordination issues varied by video type. Units featuring on-screen practitioners demonstrated stronger coherence (only 7 coordination issues, primarily vague mappings), whereas units lacking pictorial elements faced the most severe problems—41.5% showed coordination weaknesses (Figure 5).

Redundancy was most pronounced in combinations relying solely on voiceover and text, where nearly all instances suffered from replicated verbal-textual information without added semiotic value. Furthermore, repeated gestures and sound effects were frequent: identical gesture patterns appeared in 38 units, and sound repetition occurred in 31 units ([MR]), reducing overall metaphorical distinctiveness (Figure 5).

Collectively, these findings suggest that cross-modal coherence, rather than modal quantity, determines the effectiveness of metaphor representation. Overuse of redundant or weakly mapped elements diminishes communicative clarity, even when multiple modes are technically present.

## 5 Discussion

The findings reveal several key challenges in the multimodal representation of Yin Xu and Yang Xu within TCM science communication videos. These challenges center on three interrelated dimensions—modal inconsistency, imbalanced weighting, and weak cross-modal coordination—which collectively undermine the conceptual coherence of TCM metaphors. This section discusses these issues in light of the theoretical frameworks and proposes practical strategies for optimizing multimodal metaphor design.

### 5.1 Limitations in written and visual mapping

The dominance of written text and voiceover in multimodal units demonstrates that most TCM short videos remain linguistically anchored rather than semiotically integrated.

**Figure 6:**
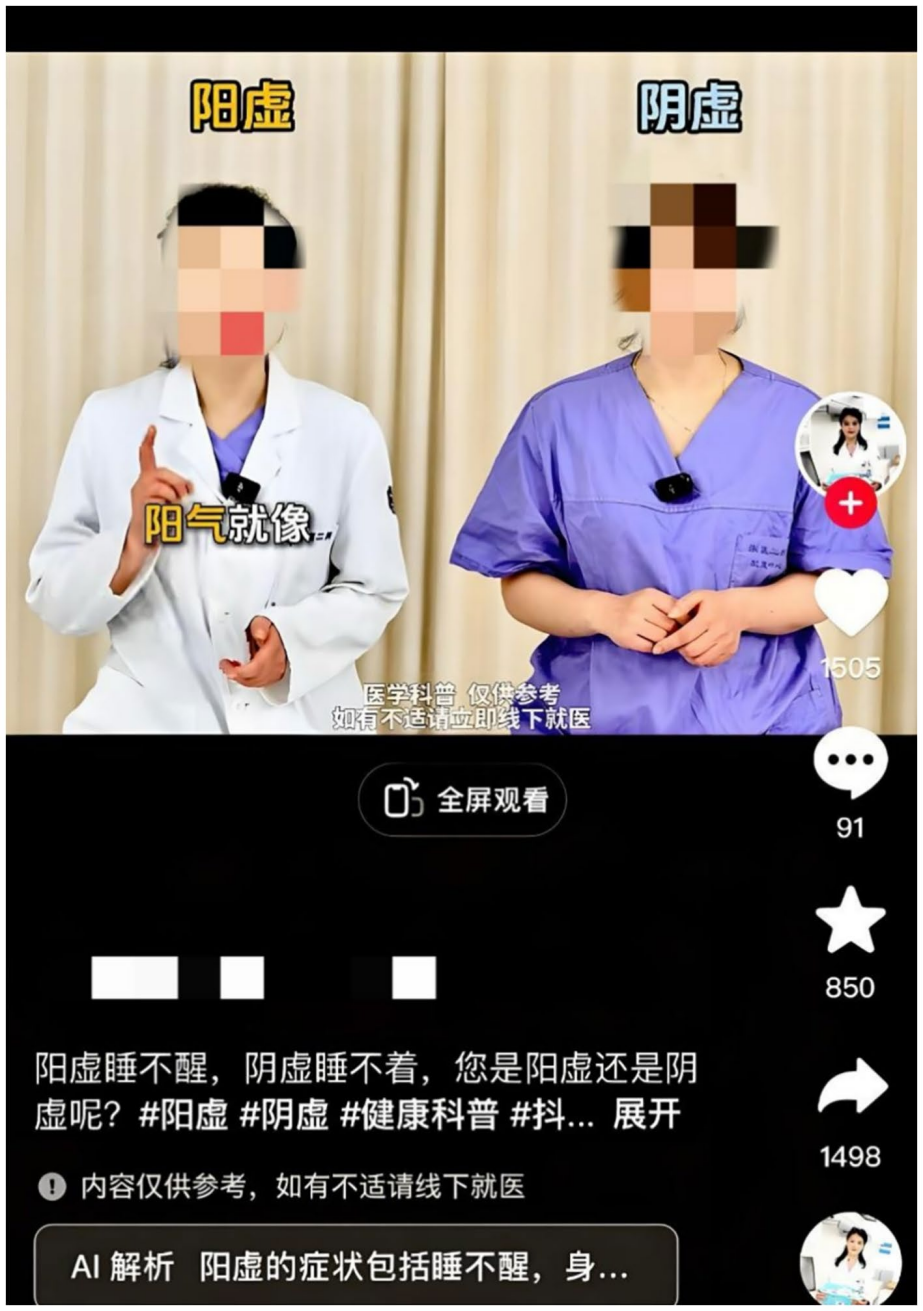
Example of contradictory font color usage in textual representations of Yin Xu and Yang Xu: blue text for Yin Xu (associated with heat syndrome) and yellow text for Yang Xu (associated with cold syndrome), directly opposing the underlying pathological logic of TCM.

However, results showed that nearly half of the textual representations conflicted with the underlying conceptual metaphors of Yin Xu and Yang Xu. For example, the frequent use of blue text for Yin Xu and red text for Yang Xu directly contradicts TCM’s metaphorical logic, in which Yin Xu is manifested by internal heat and Yang Xu by cold syndromes (see Figure 6). Such inconsistency disrupts the cognitive topology between source and target domains, leading to inferential incoherence (20).

**Figure 7:**
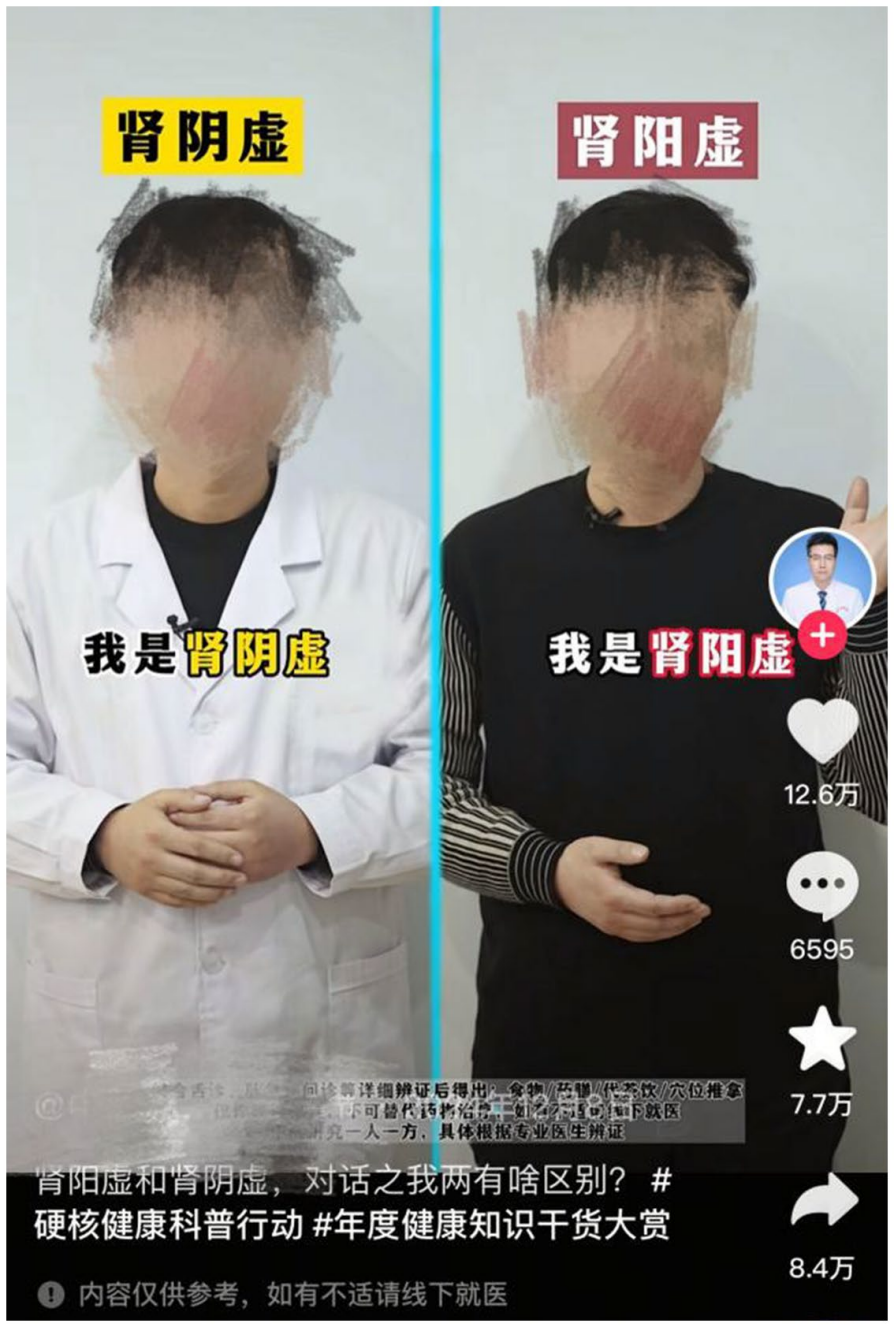
Example of ambiguous and incomplete pictorial mappings for Yin Xu and Yang Xu: a doctor wearing white clothing to represent Yin Xu (heatsyndrome) and black clothing for Yang Xu (cold syndrome), failing to align with the pathological logic of TCM.

**Figure 8:**
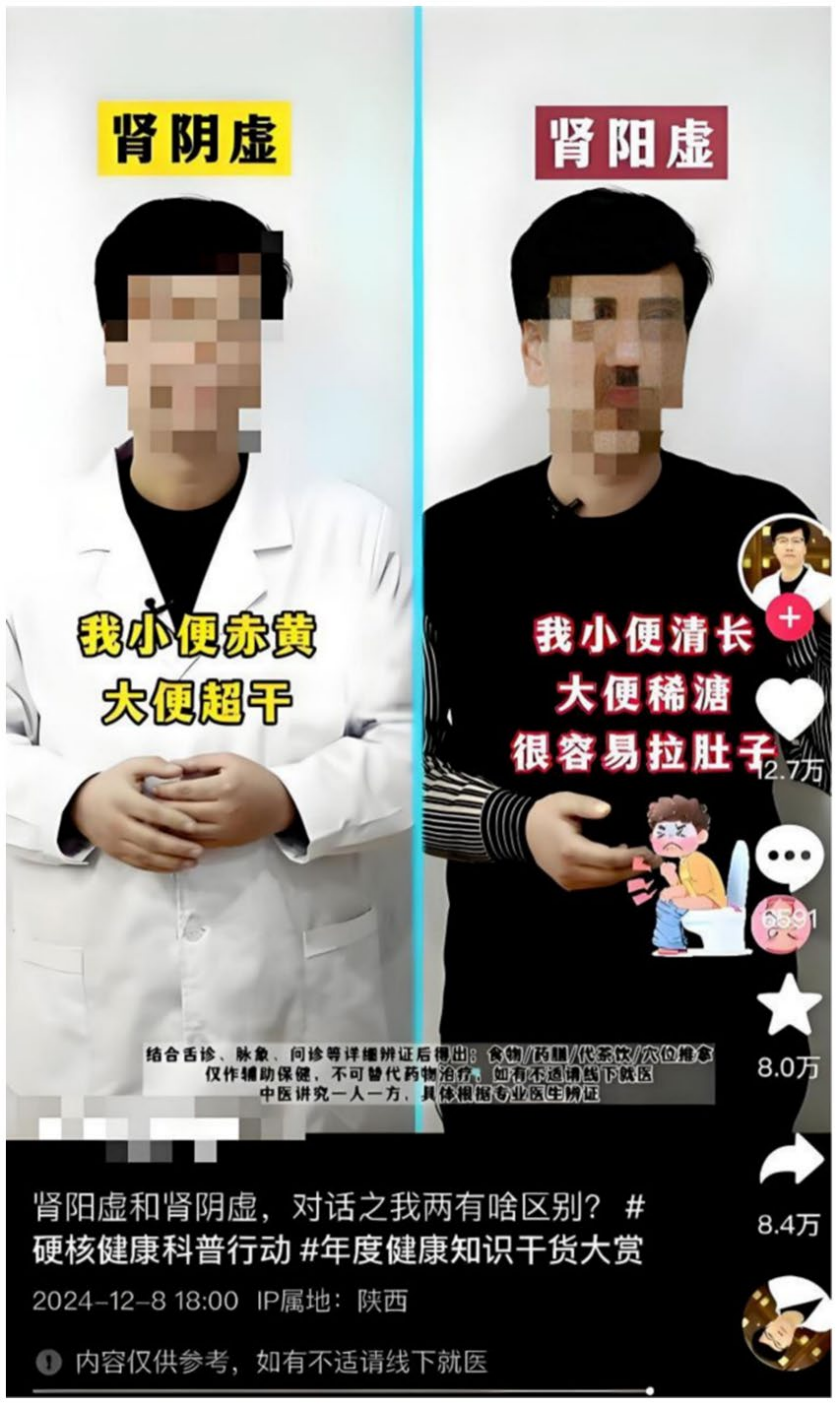
Example of ambiguous and incomplete pictorial mappings for symptoms of Yin Xu and Yang Xu: a character with a pained expression sitting on a toilet used to illustrate “Yang deficiency causes loose stools,” lacking specificity and potentially confusing viewers between “dry stools” (Yin Xu) and “loose stools” (Yang Xu).

Likewise, pictorial elements across multiple videos demonstrated inconsistent mapping and insufficient dynamic representation. When on-screen doctors embodied Yin Xu and Yang Xu, their clothing colors—blue or white for Yin Xu, purple or black for Yang Xu—mirrored the font-color problems noted earlier. These color choices failed to communicate the core pathological principles, creating contradictory visual signals instead of supporting the intended metaphorical structure (Figure 7). Additionally, visual representations often defaulted to static, standardized imagery without depicting dynamic pathological processes. For example, when illustrating “Yang deficiency causes loose stools,” certain videos displayed generic “feces” graphics or characters with pained expressions sitting on toilets. These standardized visuals created confusing connections between “dry stools” (associated with Yin Xu) and “loose stools” (associated with Yang Xu), relegating visual elements to purely decorative functions (see Figure 8). Through the lens of Visual Grammar Theory, these inaccuracies compromise symbolic attribution, while static imagery fails to provide the narrative vectors necessary for expressing causal relationships (21). This issue becomes especially problematic considering that visual components influence the perceived reliability of health content; misaligned imagery may erode trust and foster misinterpretation (5).

To enhance the effectiveness of multimodal representations, strategic chromatic coding and dynamic narrative design can be implemented across written and pictorial modes.

First, visual and textual design should be pathology-based rather than symbol-based. For instance, Yin Xu could be represented with reddish or golden hues, indicating internal dryness and heat, whereas Yang Xu should adopt pale or bluish tones, symbolizing cold stagnation. Integrating such chromatic cues into motion graphics and subtitles would strengthen the conceptual coherence of metaphors, as color serves as a key semiotic resource for encoding meaning in multimodal texts (21). Second, dynamic narratives depicting pathological processes should replace static visual content. Previous research emphasizes that narrative structures are essential for guiding viewers’ interpretation of causal and temporal relations (24). To illustrate “Yang deficiency causes clear, profuse urination and loose stools,” videos could depict a dark, rain-soaked landscape with flowing mudslides: persistent rainfall represents excessive urination, churning mudflows parallel intestinal looseness, and cloud-covered skies emphasize Yang Xu’s cold characteristics. Likewise, “Yang deficiency causes decreased libido” could combine blue typography with imagery of frozen flowers wilting on ice. These coordinated text-visual pairings serve to reduce informational load from on-screen content while creating more memorable and effective educational experiences. Through strategic distribution of meaning across complementary modes, this method alleviates perceptual strain on individual channels, enabling more fluid multimodal engagement (25).

### 5.2 Weaknesses in auditory and gestural representation

The results also indicated that although auditory and gestural modes were present, they were often poorly synchronized with the verbal meaning and lacked interpretive clarity. More than 70% of the sound effects showed either inaccurate or incomplete mapping of the source domain. First, sound mappings often failed to align with the pathological logic of the target domain. For example, pairing a “farting” sound effect with the statement “Yin deficiency causes dry stools” produced a misleading association, since flatulence is more typically linked to cold-related digestive disturbances rather than the heat-related dryness associated with Yin Xu. Second, sound mappings were frequently incomplete, providing only partial support for the metaphor. A typical example was the use of “howling wind” audio to mirror “cold aversion due to Yang deficiency.” While the wind sound effectively evoked the cold nature of Yang Xu, it failed to suggest any corresponding bodily manifestation, leaving the metaphor underdetermined.

**Figure 9:**
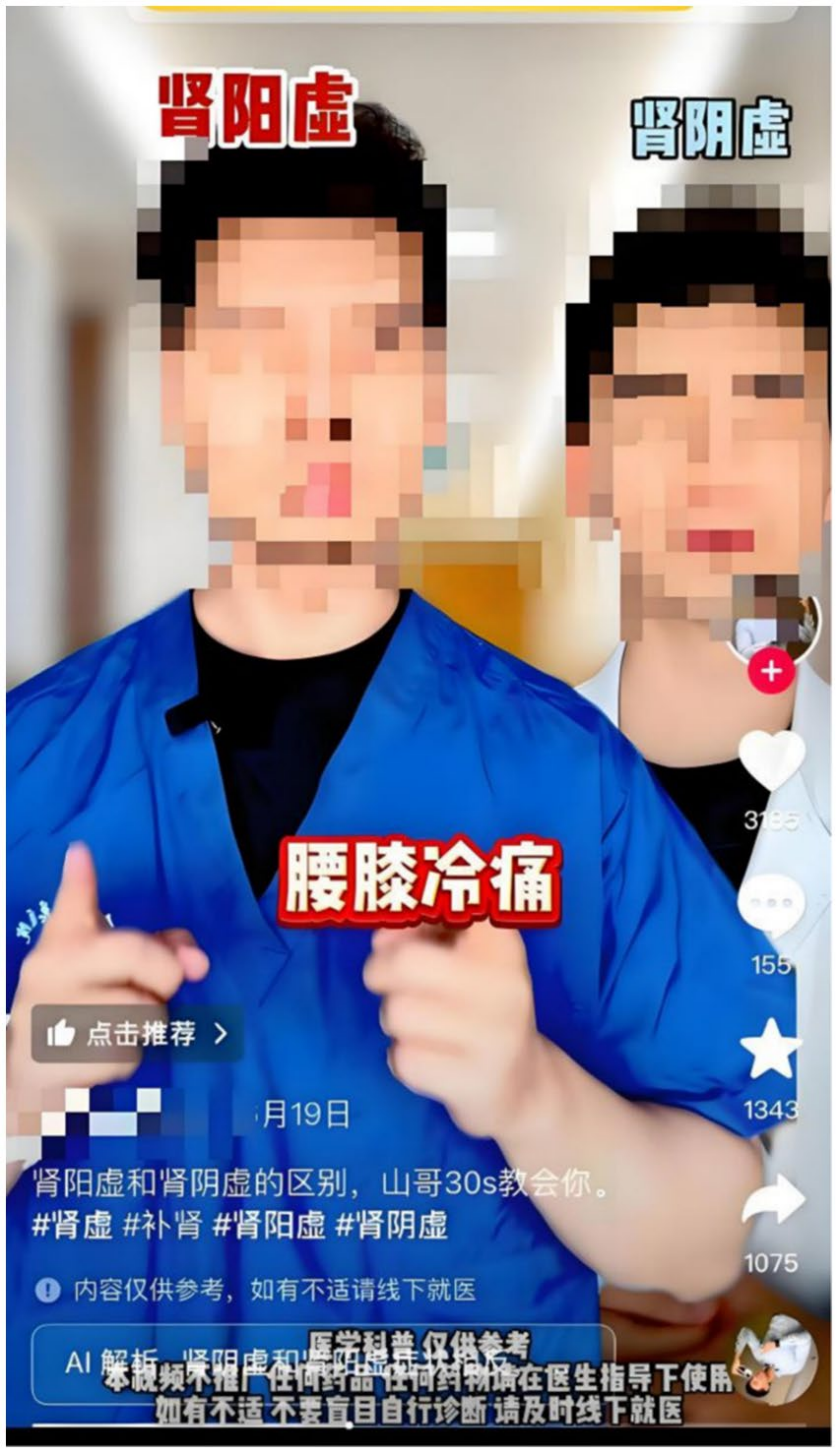
Example of vague and redundant gestural mappings for symptoms of Yin Xu and Yang Xu. The on-screen doctor uses identical finger-waving gestures to illustrate both “Yin deficiency causes high libido” and “Yang deficiency causes low libido,” failing to encode the critical distinction between the two opposing concepts. This vague gesture neutralizes the embodied metaphor, reducing it to mere ornamentation.

Gestures, meanwhile, frequently functioned as repetitive or ornamental movements rather than meaningful semiotic resources. This inconsistency in gestural mapping largely stemmed from redundancy rooted in individual doctors’ habitual body language. Repeated hand-waving synchronized with voiceovers introduced irrelevant associations, risking distracting viewers from key information. For example, in segments illustrating “Yin deficiency causes high libido, whereas Yang deficiency causes low libido,” some doctors used identical finger-waving gestures for both syndromes, failing to represent the critical distinction in libido levels (see Figure 9). Given that gestures play a crucial role in encoding conceptual distinctions (26), deploying identical gestures for opposing concepts neutralizes this embodied metaphor and reduces gesture to mere ornamentation.

To optimize non-verbal representation, each mode should be semantically anchored in the pathological logic of the metaphor, thereby ensuring that each symptom is paired with distinct cues to avoid confusing associations. For auditory cues, a “gurgling water” sound effect can represent “Yang deficiency causes loose stools,” reflecting the cold-induced dampness characteristic of Yang Xu. Conversely, a crackling sound effect can accompany “Yin deficiency causes dry stools,” corresponding to the dryness resulting from fluid depletion in Yin Xu. For gestural cues, fast, upward movements paired with sizzling sound effects can reinforce the restlessness and heat associated with Yin Xu. In contrast, slow, inward gestures combined with wind-like or low-frequency sounds can embody the cold aversion and listlessness typical of Yang Xu. These strategies not only align with the inferential structure of TCM metaphors but also strengthen embodied cognition, enabling audiences to experience the metaphor rather than merely decode it (27).

### 5.3 Cross-modal coordination and redundancy

Although the rate of cross-modal asynchrony was relatively low, nearly half of the metaphorical units exhibited weak coordination between modes. Excessive duplication—such as presenting identical content simultaneously in voiceover and on-screen text—leads to redundant salience. According to Multimodal Interaction Analysis, effective multimodal communication requires functional complementarity rather than mere simultaneity. To address this, multimodal design should aim for cross-modal synergy, in which each mode amplifies rather than merely replicates the others.

Consider the illustration of libido differences: “Yin deficiency causes high libido” could be represented by an animated hot-air balloon rising with flames beneath it, accompanied by a “whoosh” sound effect and a palm-raising gesture. In contrast, “Yang deficiency causes low libido” might be depicted by a balloon drifting downward amid wind and rain, paired with a low-battery alert sound and a palm-lowering gesture. Anchored by voiceover narration, such coordinated multimodal cues render abstract pathological relationships more tangible and accessible. Effective multimodal communication depends on careful modal coordination, as dissonance across modes can disrupt metaphorical coherence and undermine the credibility of information (25).

### 5.4 Implications for digital science communication

The findings hold broader implications for digital health and science communication beyond TCM. They demonstrate that multimodal richness does not automatically equate to conceptual coherence. Without conceptual coherence, even visually appealing short videos risk devolving into esthetic noise—attractive but semantically shallow (28). Incorporating dynamic narrative strategies—such as animated progression from symptom to recovery—could better reflect the cyclical balance central to TCM philosophy (24). Moreover, systematic design grounded in multimodal metaphor principles can transform digital TCM communication from didactic narration into embodied learning experiences (20, 27). Such improvements may strengthen both public understanding of TCM concepts and cross-cultural communication of TCM knowledge in the global context (29).

## 6 Conclusion

This study examined how multimodal metaphors represent the TCM concepts of Yin Xu and Yang Xu in Douyin short videos. Through a systematic multimodal annotation of 89 metaphorical units across 15 videos, the research identified significant inconsistencies, imbalanced modal weighting, and weak cross-modal coordination as the primary obstacles to accurate digital representation of TCM metaphors.

### 6.1 Summary of key findings

First, the dominance of textual and verbal modes resulted in linguistically overloaded but semiotically shallow representations. Over 40% of written text exhibited inconsistent color or lexical mapping with the conceptual logic of Yin Xu and Yang Xu.

Second, pictorial, auditory, and gestural modes were often underutilized or only loosely connected to the target domain, often serving decorative rather than cognitive functions.

Third, half of all multimodal units demonstrated weak complementarity, with redundant or conflicting mappings across modes, thereby diminishing overall metaphorical clarity.

Collectively, these findings suggest that the quality of cross-modal coherence, rather than the number of modal accuracy, determines the communicative effectiveness of TCM digital metaphors.

### 6.2 Theoretical and practical implications

Theoretically, this study extends Conceptual Metaphor Theory and Multimodal Metaphor Theory into the domain of digital health communication, demonstrating how cognitive metaphorical logic can be operationalized and quantitatively measured in audiovisual contexts.

By integrating Visual Grammar Theory, it also provides a framework for evaluating how visual and auditory salience interact with linguistic anchoring to shape public interpretation.

Practically, the findings offer clear design implications for digital TCM dissemination: metaphor visualization should adhere to pathological logic, not merely symbolic esthetics; dynamic narratives—such as temperature transition or bodily change animations—should replace static visuals; cross-modal coordination should prioritize semantic complementarity, ensuring that each mode contributes unique meaning rather than redundant duplication.

These strategies can help transform TCM short videos from visually attractive yet conceptually fragmented products into coherent and authentic science communication tools.

### 6.3 Limitations and future research

Despite rigorous annotation and quantitative design, this study has several limitations. First, given its exploratory nature, the analysis relies primarily on descriptive statistics to identify patterns rather than on inferential testing to establish causal relationships. While this approach aligns with the study’s current objectives, it limits the ability to draw generalizable causal conclusions. Second, the relatively small sample of 15 videos restricts the extent to which the findings can be generalized to broader contexts of TCM communication. Third, while annotation subjectivity was minimized through high inter-coder reliability (*κ* = 0.86), some degree of interpretive bias may persist. Future research could address this by involving a larger and more diverse panel of annotators, including both multimodal analysts and TCM specialists. Finally, this study analyzes multimodal representations themselves but does not empirically test their communicative effectiveness. Therefore, future studies should incorporate larger, multi-platform datasets and utilize experimental methodologies including eye-tracking or EEG to examine how multimodal coherence affects audience cognition and learning outcomes (28, 30). Interdisciplinary collaboration among TCM specialists, cognitive linguists, and digital designers could further yield more sophisticated and empirically robust multimodal frameworks for science communication.

### 6.4 Concluding remarks

Metaphor is the cognitive foundation through which TCM conceptualizes the body, illness, and treatment. When translated into digital multimodal environments, these metaphors risk losing their explanatory power if reduced to superficial rhetorical symbols. By systematically linking multimodal semiotic analysis with TCM’s inherent metaphorical reasoning, this study demonstrates how ancient conceptual frameworks can be revitalized through modern media design. Ultimately, integrating traditional knowledge with multimodal communication principles not only advances the modernization of TCM but also helps bridge Eastern and Western medical paradigms.

## Data Availability

The datasets presented in this study can be found in online repositories. The names of the repository/repositories and accession number(s) can be found below: https://doi.org/10.6084/m9.figshare.29926961.

## Author’s note

Taiji Diagram (太极图), commonly known as the Yin-Yang symbol, is an ancient Chinese representation of complementary opposites (Yin and Yang) in harmonious balance, illustrating the dynamic interdependence and cyclical transformation of all natural forces.
